# *N*′-[1-(4-Chloro­phen­yl)ethyl­idene]-5-methyl-1-(4-nitro­phen­yl)-1*H*-1,2,3-triazole-4-carbohydrazide

**DOI:** 10.1107/S1600536812027535

**Published:** 2012-06-23

**Authors:** Hoong-Kun Fun, Ching Kheng Quah, Nitinchandra, Balakrishna Kalluraya, Shobhitha Shetty

**Affiliations:** a X-ray Crystallography Unit, School of Physics, Universiti Sains Malaysia, 11800 USM, Penang, Malaysia; b Department of Studies in Chemistry, Mangalore University, Mangalagangotri, Mangalore 574 199, India

## Abstract

In the title compound, C_18_H_15_ClN_6_O_3_, the 1,2,3-triazole ring forms dihedral angles of 15.64 (5) and 57.50 (5)° with the two benzene rings. The dihedral angle between the two benzene rings is 72.26 (5)°. In the crystal, mol­ecules are linked *via* C—H⋯O hydrogen bonds into chains propagating along the *b* axis. A short O⋯C contact of 2.9972 (13) Å is observed.

## Related literature

For general background to and the biological activity of triazole derivatives, see: Sherement *et al.* (2004 ▶); Danoun *et al.* (1998 ▶); Manfredini *et al.* (2000 ▶); Biagi *et al.* (2004 ▶); Vijayakumar *et al.* (2011 ▶). For standard bond-length data, see: Allen *et al.* (1987 ▶). For the stability of the temperature controller used in the data collection, see: Cosier & Glazer (1986 ▶). For related structures, see: Fun, Quah, Chandrakantha *et al.* (2011 ▶); Fun *et al.* (2011*a* ▶,*b* ▶).
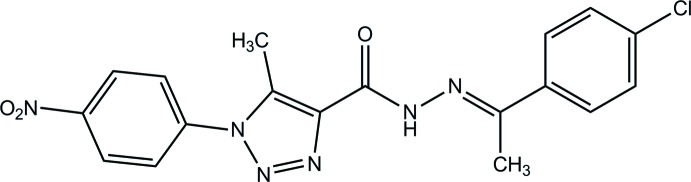


## Experimental

### 

#### Crystal data

C_18_H_15_ClN_6_O_3_
*M* *_r_* = 398.81
Triclinic, 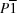

*a* = 8.6603 (1) Å
*b* = 10.2844 (1) Å
*c* = 10.4033 (1) Å
α = 83.816 (1)°
β = 81.402 (1)°
γ = 76.373 (1)°
*V* = 887.84 (2) Å^3^
*Z* = 2
Mo *K*α radiation
μ = 0.25 mm^−1^
*T* = 100 K
0.31 × 0.19 × 0.17 mm

#### Data collection

Bruker SMART APEXII CCD area-detector diffractometer
Absorption correction: multi-scan (*SADABS*; Bruker, 2009 ▶) *T*_min_ = 0.927, *T*_max_ = 0.958
29293 measured reflections
7839 independent reflections
6598 reflections with *I* > 2σ(*I*)
*R*_int_ = 0.023

#### Refinement

*R*[*F* ^2^ > 2σ(*F* ^2^)] = 0.041
*wR*(*F* ^2^) = 0.120
*S* = 1.03
7839 reflections
259 parameters
H atoms treated by a mixture of independent and constrained refinement
Δρ_max_ = 0.56 e Å^−3^
Δρ_min_ = −0.45 e Å^−3^

### 

Data collection: *APEX2* (Bruker, 2009 ▶); cell refinement: *SAINT* (Bruker, 2009 ▶); data reduction: *SAINT*; program(s) used to solve structure: *SHELXTL* (Sheldrick, 2008 ▶); program(s) used to refine structure: *SHELXTL*; molecular graphics: *SHELXTL*; software used to prepare material for publication: *SHELXTL* and *PLATON* (Spek, 2009 ▶).

## Supplementary Material

Crystal structure: contains datablock(s) global, I. DOI: 10.1107/S1600536812027535/is5158sup1.cif

Structure factors: contains datablock(s) I. DOI: 10.1107/S1600536812027535/is5158Isup2.hkl

Supplementary material file. DOI: 10.1107/S1600536812027535/is5158Isup3.cml

Additional supplementary materials:  crystallographic information; 3D view; checkCIF report

## Figures and Tables

**Table 1:** Hydrogen-bond geometry (Å, °)

*D*—H⋯*A*	*D*—H	H⋯*A*	*D*⋯*A*	*D*—H⋯*A*
C13—H13*A*⋯O1^i^	0.95	2.32	3.2226 (12)	158

Symmetry code: (i) 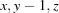.

**Fig. 1.**
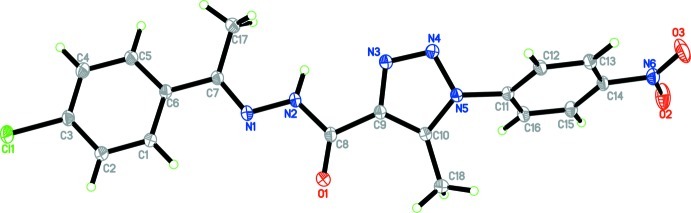
The molecular structure of the title compound showing 50% probability displacement ellipsoids for non-H atoms.

**Fig. 2.**
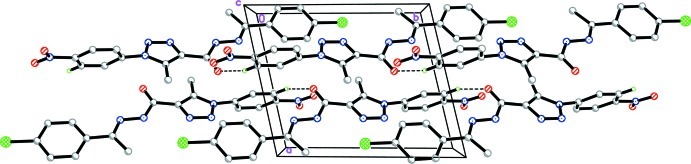
A packing diagram of the title compound, viewed along the c axis. H atoms not involved in hydrogen bonds (dashed lines) have been omitted for clarity.

## supplementary crystallographic information

### Comment

1,2,3-Triazole and its derivatives had attracted considerable attention for the past few decades due to their chemotherapeutic value. Many 1,2,3-triazoles are found to be potent antimicrobial (Sherement *et al.*, 2004) and antiviral agents. Some of them have exhibited antiproliferative and anticancer activities (Danoun *et al.*, 1998). Some 1,2,3-triazoles are used as DNA cleaving agents (Manfredini *et al.*, 2000) and potassium channel activators (Biagi *et al.*, 2004). Hydrazones derived from anisaldehyde and 4-nitro-5-ethoxycarbonyl phenylhydrazine showed excellent NLO property (Vijayakumar *et al.*, 2011). Prompted by these observation, we synthesized new hydrazone carrying 1,2,3-triazoles nucleus.

In the title molecule, Fig. 1, the mean plane of 1,2,3-triazole ring (N3-N5/C9/C10, r.m.s deviation = 0.002 Å) forms dihedral angles of 15.64 (5) and 57.50 (5)° with the two benzene rings (C1–C6 and C11–C16). The dihedral angle between the two benzenel rings is 72.26 (5)°. Bond lengths (Allen *et al.*, 1987) and angles are within normal ranges and are comparable to related structures (Fun, Quah, Chandrakantha *et al.*, 2011; Fun *et al.*, 2011*a*,*b*). In the crystal (Fig. 2), molecules are linked *via* intermolecular C13—H13A···O1 hydrogen bonds (Table 1) into chains propagating along [010]. A short O2···C7 contact of 2.9972 (13) Å also occurs.

### Experimental

The title compound was obtained by refluxing a mixture of 5-methyl-1-(4-nitrophenyl)-1*H*-1,2,3-triazole-4-carbohydrazide (0.01 mol) and p-chloroacetophenone (0.01 mol) in ethanol (30 ml) and 3 drops of concentrated sulfuric acid for 1 h. Excess ethanol was removed from the reaction mixture under reduced pressure. The solid product obtained was filtered, washed with ethanol and dried. Single crystals suitable for X-ray analysis were obtained by slow evaporation of an ethanol-*N*,*N*-dimethylformamide (DMF) (3:1) solution.

### Refinement

Atom H1N2 was located in a difference Fourier map and refined freely [N—H = 0.870 (18) Å]. The rest of hydrogen atoms were positioned geometrically and refined using a riding model with C—H = 0.95 or 0.98 Å and with *U*_iso_(H) = 1.2 or 1.5*U*_eq_(C). A rotating-group model was applied for the methyl groups.

### Crystal data

**Table:**

C_18_H_15_ClN_6_O_3_	*Z* = 2
*M**_r_* = 398.81	*F*(000) = 412
Triclinic, *P*1	*D*_x_ = 1.492 Mg m^−^^3^
Hall symbol: -P 1	Mo *K*α radiation, λ = 0.71073 Å
*a* = 8.6603 (1) Å	Cell parameters from 9924 reflections
*b* = 10.2844 (1) Å	θ = 2.4–35.1°
*c* = 10.4033 (1) Å	µ = 0.25 mm^−^^1^
α = 83.816 (1)°	*T* = 100 K
β = 81.402 (1)°	Block, yellow
γ = 76.373 (1)°	0.31 × 0.19 × 0.17 mm
*V* = 887.84 (2) Å^3^	

### Data collection

**Table:**

Bruker SMART APEXII CCD area-detector diffractometer	7839 independent reflections
Radiation source: fine-focus sealed tube	6598 reflections with *I* > 2σ(*I*)
Graphite monochromator	*R*_int_ = 0.023
φ and ω scans	θ_max_ = 35.2°, θ_min_ = 2.0°
Absorption correction: multi-scan (*SADABS*; Bruker, 2009)	*h* = −14→13
*T*_min_ = 0.927, *T*_max_ = 0.958	*k* = −16→16
29293 measured reflections	*l* = −16→16

### Refinement

**Table:**

Refinement on *F*^2^	Primary atom site location: structure-invariant direct methods
Least-squares matrix: full	Secondary atom site location: difference Fourier map
*R*[*F*^2^ > 2σ(*F*^2^)] = 0.041	Hydrogen site location: inferred from neighbouring sites
*wR*(*F*^2^) = 0.120	H atoms treated by a mixture of independent and constrained refinement
*S* = 1.03	*w* = 1/[σ^2^(*F*_o_^2^) + (0.0635*P*)^2^ + 0.2969*P*] where *P* = (*F*_o_^2^ + 2*F*_c_^2^)/3
7839 reflections	(Δ/σ)_max_ = 0.001
259 parameters	Δρ_max_ = 0.56 e Å^−^^3^
0 restraints	Δρ_min_ = −0.45 e Å^−^^3^

### Special details

**Table:**

Experimental. The crystal was placed in the cold stream of an Oxford Cryosystems Cobra open-flow nitrogen cryostat (Cosier & Glazer, 1986) operating at 100.0 (1) K.
Geometry. All esds (except the esd in the dihedral angle between two l.s. planes) are estimated using the full covariance matrix. The cell esds are taken into account individually in the estimation of esds in distances, angles and torsion angles; correlations between esds in cell parameters are only used when they are defined by crystal symmetry. An approximate (isotropic) treatment of cell esds is used for estimating esds involving l.s. planes.
Refinement. Refinement of F^2^ against ALL reflections. The weighted R-factor wR and goodness of fit S are based on F^2^, conventional R-factors R are based on F, with F set to zero for negative F^2^. The threshold expression of F^2^ > 2sigma(F^2^) is used only for calculating R-factors(gt) etc. and is not relevant to the choice of reflections for refinement. R-factors based on F^2^ are statistically about twice as large as those based on F, and R- factors based on ALL data will be even larger.

### Fractional atomic coordinates and isotropic or equivalent isotropic displacement parameters (Å^2^)

**Table:**

	*x*	*y*	*z*	*U*_iso_*/*U*_eq_	
Cl1	0.07731 (3)	1.51350 (2)	0.15822 (3)	0.02455 (7)	
O1	0.43782 (9)	0.74131 (7)	0.59589 (7)	0.01901 (13)	
O2	0.30629 (12)	−0.13446 (9)	1.10163 (8)	0.0326 (2)	
O3	0.38384 (15)	−0.24022 (8)	0.92733 (9)	0.0389 (2)	
N1	0.21957 (10)	0.88716 (7)	0.43384 (8)	0.01537 (13)	
N2	0.23356 (10)	0.75384 (8)	0.47404 (8)	0.01648 (14)	
N3	0.23287 (10)	0.49322 (8)	0.53284 (8)	0.01770 (14)	
N4	0.23488 (11)	0.37257 (8)	0.58529 (8)	0.01845 (15)	
N5	0.32874 (10)	0.35228 (7)	0.68349 (7)	0.01458 (13)	
N6	0.34560 (11)	−0.13715 (8)	0.98386 (9)	0.01954 (15)	
C1	0.20505 (11)	1.15159 (9)	0.34403 (9)	0.01614 (15)	
H1A	0.2840	1.1076	0.3981	0.019*	
C2	0.19901 (12)	1.28429 (9)	0.29798 (9)	0.01779 (16)	
H2A	0.2732	1.3307	0.3197	0.021*	
C3	0.08252 (12)	1.34817 (9)	0.21943 (9)	0.01661 (15)	
C4	−0.02861 (12)	1.28298 (9)	0.18865 (10)	0.01829 (16)	
H4A	−0.1087	1.3283	0.1362	0.022*	
C5	−0.02088 (11)	1.14978 (9)	0.23606 (9)	0.01716 (15)	
H5A	−0.0974	1.1048	0.2161	0.021*	
C6	0.09732 (10)	1.08100 (9)	0.31252 (8)	0.01409 (14)	
C7	0.11168 (11)	0.93734 (9)	0.35781 (9)	0.01475 (14)	
C8	0.34084 (11)	0.69073 (8)	0.55704 (8)	0.01471 (14)	
C9	0.32483 (11)	0.55123 (8)	0.59531 (8)	0.01430 (14)	
C10	0.38769 (11)	0.46173 (9)	0.69309 (9)	0.01486 (15)	
C11	0.34388 (11)	0.22838 (8)	0.76168 (8)	0.01399 (14)	
C12	0.40005 (11)	0.10949 (9)	0.70018 (9)	0.01562 (15)	
H12A	0.4375	0.1114	0.6095	0.019*	
C13	0.40066 (11)	−0.01205 (9)	0.77318 (9)	0.01625 (15)	
H13A	0.4358	−0.0947	0.7334	0.019*	
C14	0.34846 (11)	−0.00917 (9)	0.90596 (9)	0.01519 (15)	
C15	0.29653 (12)	0.10859 (9)	0.96930 (9)	0.01683 (15)	
H15A	0.2643	0.1063	1.0607	0.020*	
C16	0.29298 (11)	0.22988 (9)	0.89527 (9)	0.01648 (15)	
H16A	0.2565	0.3125	0.9350	0.020*	
C17	0.00858 (14)	0.85722 (11)	0.31287 (12)	0.0258 (2)	
H17A	0.0763	0.7751	0.2777	0.039*	
H17B	−0.0510	0.9106	0.2449	0.039*	
H17C	−0.0669	0.8336	0.3867	0.039*	
C18	0.49787 (15)	0.46863 (11)	0.78744 (11)	0.0260 (2)	
H18A	0.5705	0.3807	0.7998	0.039*	
H18B	0.4354	0.4940	0.8711	0.039*	
H18C	0.5604	0.5357	0.7537	0.039*	
H1N2	0.166 (2)	0.7102 (18)	0.4563 (17)	0.036 (4)*	

### Atomic displacement parameters (Å^2^)

**Table:**

	*U*^11^	*U*^22^	*U*^33^	*U*^12^	*U*^13^	*U*^23^
Cl1	0.03489 (14)	0.01270 (10)	0.02708 (12)	−0.00533 (8)	−0.01090 (10)	0.00374 (8)
O1	0.0224 (3)	0.0150 (3)	0.0216 (3)	−0.0064 (2)	−0.0073 (3)	0.0008 (2)
O2	0.0440 (5)	0.0211 (4)	0.0239 (4)	−0.0016 (3)	0.0085 (3)	0.0065 (3)
O3	0.0768 (8)	0.0139 (3)	0.0274 (4)	−0.0118 (4)	−0.0103 (4)	0.0005 (3)
N1	0.0177 (3)	0.0118 (3)	0.0165 (3)	−0.0038 (2)	−0.0032 (3)	0.0019 (2)
N2	0.0197 (3)	0.0118 (3)	0.0190 (3)	−0.0052 (3)	−0.0061 (3)	0.0030 (2)
N3	0.0223 (4)	0.0151 (3)	0.0175 (3)	−0.0071 (3)	−0.0062 (3)	0.0025 (3)
N4	0.0239 (4)	0.0161 (3)	0.0177 (3)	−0.0078 (3)	−0.0080 (3)	0.0032 (3)
N5	0.0180 (3)	0.0123 (3)	0.0144 (3)	−0.0048 (2)	−0.0041 (2)	0.0008 (2)
N6	0.0214 (4)	0.0149 (3)	0.0222 (4)	−0.0047 (3)	−0.0045 (3)	0.0032 (3)
C1	0.0186 (4)	0.0148 (3)	0.0159 (3)	−0.0048 (3)	−0.0054 (3)	0.0018 (3)
C2	0.0225 (4)	0.0148 (3)	0.0177 (4)	−0.0060 (3)	−0.0061 (3)	0.0006 (3)
C3	0.0208 (4)	0.0115 (3)	0.0166 (4)	−0.0023 (3)	−0.0031 (3)	0.0006 (3)
C4	0.0178 (4)	0.0159 (4)	0.0203 (4)	−0.0016 (3)	−0.0055 (3)	0.0019 (3)
C5	0.0156 (4)	0.0161 (4)	0.0201 (4)	−0.0041 (3)	−0.0045 (3)	0.0018 (3)
C6	0.0143 (3)	0.0132 (3)	0.0143 (3)	−0.0033 (3)	−0.0014 (3)	0.0009 (3)
C7	0.0149 (3)	0.0139 (3)	0.0156 (3)	−0.0046 (3)	−0.0023 (3)	0.0016 (3)
C8	0.0177 (4)	0.0120 (3)	0.0138 (3)	−0.0031 (3)	−0.0016 (3)	0.0006 (3)
C9	0.0166 (3)	0.0121 (3)	0.0145 (3)	−0.0040 (3)	−0.0029 (3)	0.0007 (3)
C10	0.0172 (4)	0.0119 (3)	0.0160 (3)	−0.0039 (3)	−0.0037 (3)	0.0002 (3)
C11	0.0155 (3)	0.0119 (3)	0.0147 (3)	−0.0040 (3)	−0.0025 (3)	0.0011 (3)
C12	0.0182 (4)	0.0138 (3)	0.0149 (3)	−0.0039 (3)	−0.0016 (3)	−0.0009 (3)
C13	0.0186 (4)	0.0127 (3)	0.0175 (4)	−0.0034 (3)	−0.0028 (3)	−0.0007 (3)
C14	0.0159 (3)	0.0120 (3)	0.0176 (4)	−0.0038 (3)	−0.0029 (3)	0.0020 (3)
C15	0.0193 (4)	0.0148 (3)	0.0151 (3)	−0.0032 (3)	−0.0004 (3)	0.0007 (3)
C16	0.0202 (4)	0.0127 (3)	0.0155 (4)	−0.0027 (3)	−0.0008 (3)	−0.0007 (3)
C17	0.0286 (5)	0.0222 (4)	0.0321 (5)	−0.0143 (4)	−0.0149 (4)	0.0086 (4)
C18	0.0358 (6)	0.0183 (4)	0.0299 (5)	−0.0112 (4)	−0.0197 (4)	0.0046 (4)

### Geometric parameters (Å, º)

**Table:**

Cl1—C3	1.7429 (9)	C5—H5A	0.9500
O1—C8	1.2214 (11)	C6—C7	1.4836 (12)
O2—N6	1.2231 (12)	C7—C17	1.4998 (14)
O3—N6	1.2205 (12)	C8—C9	1.4799 (12)
N1—C7	1.2926 (12)	C9—C10	1.3806 (12)
N1—N2	1.3722 (10)	C10—C18	1.4852 (13)
N2—C8	1.3657 (12)	C11—C12	1.3915 (12)
N2—H1N2	0.870 (18)	C11—C16	1.3943 (12)
N3—N4	1.2979 (11)	C12—C13	1.3903 (12)
N3—C9	1.3692 (12)	C12—H12A	0.9500
N4—N5	1.3676 (11)	C13—C14	1.3889 (13)
N5—C10	1.3594 (11)	C13—H13A	0.9500
N5—C11	1.4257 (11)	C14—C15	1.3892 (13)
N6—C14	1.4728 (12)	C15—C16	1.3900 (12)
C1—C2	1.3884 (12)	C15—H15A	0.9500
C1—C6	1.4025 (13)	C16—H16A	0.9500
C1—H1A	0.9500	C17—H17A	0.9800
C2—C3	1.3912 (13)	C17—H17B	0.9800
C2—H2A	0.9500	C17—H17C	0.9800
C3—C4	1.3859 (14)	C18—H18A	0.9800
C4—C5	1.3948 (13)	C18—H18B	0.9800
C4—H4A	0.9500	C18—H18C	0.9800
C5—C6	1.4007 (13)		
			
C7—N1—N2	115.65 (8)	N3—C9—C10	109.28 (8)
C8—N2—N1	120.88 (8)	N3—C9—C8	120.79 (8)
C8—N2—H1N2	117.8 (12)	C10—C9—C8	129.91 (8)
N1—N2—H1N2	120.8 (12)	N5—C10—C9	103.05 (8)
N4—N3—C9	109.30 (8)	N5—C10—C18	124.22 (8)
N3—N4—N5	106.77 (7)	C9—C10—C18	132.69 (9)
C10—N5—N4	111.60 (7)	C12—C11—C16	122.23 (8)
C10—N5—C11	130.38 (8)	C12—C11—N5	118.44 (8)
N4—N5—C11	117.94 (7)	C16—C11—N5	119.21 (8)
O3—N6—O2	123.62 (9)	C13—C12—C11	119.11 (8)
O3—N6—C14	118.11 (9)	C13—C12—H12A	120.4
O2—N6—C14	118.27 (8)	C11—C12—H12A	120.4
C2—C1—C6	121.50 (8)	C14—C13—C12	118.08 (8)
C2—C1—H1A	119.3	C14—C13—H13A	121.0
C6—C1—H1A	119.3	C12—C13—H13A	121.0
C1—C2—C3	118.88 (9)	C13—C14—C15	123.40 (8)
C1—C2—H2A	120.6	C13—C14—N6	118.58 (8)
C3—C2—H2A	120.6	C15—C14—N6	118.02 (8)
C4—C3—C2	121.41 (8)	C14—C15—C16	118.22 (8)
C4—C3—Cl1	119.51 (7)	C14—C15—H15A	120.9
C2—C3—Cl1	119.08 (7)	C16—C15—H15A	120.9
C3—C4—C5	118.87 (8)	C15—C16—C11	118.92 (8)
C3—C4—H4A	120.6	C15—C16—H16A	120.5
C5—C4—H4A	120.6	C11—C16—H16A	120.5
C4—C5—C6	121.37 (9)	C7—C17—H17A	109.5
C4—C5—H5A	119.3	C7—C17—H17B	109.5
C6—C5—H5A	119.3	H17A—C17—H17B	109.5
C5—C6—C1	117.93 (8)	C7—C17—H17C	109.5
C5—C6—C7	121.78 (8)	H17A—C17—H17C	109.5
C1—C6—C7	120.28 (8)	H17B—C17—H17C	109.5
N1—C7—C6	115.96 (8)	C10—C18—H18A	109.5
N1—C7—C17	123.38 (8)	C10—C18—H18B	109.5
C6—C7—C17	120.63 (8)	H18A—C18—H18B	109.5
O1—C8—N2	125.21 (8)	C10—C18—H18C	109.5
O1—C8—C9	123.63 (8)	H18A—C18—H18C	109.5
N2—C8—C9	111.15 (8)	H18B—C18—H18C	109.5
			
C7—N1—N2—C8	−178.43 (8)	N2—C8—C9—C10	−167.16 (9)
C9—N3—N4—N5	0.29 (10)	N4—N5—C10—C9	0.09 (10)
N3—N4—N5—C10	−0.24 (11)	C11—N5—C10—C9	−176.51 (9)
N3—N4—N5—C11	176.83 (8)	N4—N5—C10—C18	−177.85 (10)
C6—C1—C2—C3	0.35 (14)	C11—N5—C10—C18	5.55 (16)
C1—C2—C3—C4	1.24 (14)	N3—C9—C10—N5	0.09 (10)
C1—C2—C3—Cl1	−178.52 (7)	C8—C9—C10—N5	178.30 (9)
C2—C3—C4—C5	−1.10 (14)	N3—C9—C10—C18	177.77 (11)
Cl1—C3—C4—C5	178.66 (7)	C8—C9—C10—C18	−4.03 (18)
C3—C4—C5—C6	−0.64 (14)	C10—N5—C11—C12	−126.78 (10)
C4—C5—C6—C1	2.15 (14)	N4—N5—C11—C12	56.80 (12)
C4—C5—C6—C7	−176.84 (9)	C10—N5—C11—C16	57.15 (13)
C2—C1—C6—C5	−2.00 (14)	N4—N5—C11—C16	−119.27 (10)
C2—C1—C6—C7	177.00 (8)	C16—C11—C12—C13	2.20 (14)
N2—N1—C7—C6	−179.22 (7)	N5—C11—C12—C13	−173.74 (8)
N2—N1—C7—C17	−1.02 (14)	C11—C12—C13—C14	−1.61 (13)
C5—C6—C7—N1	−176.20 (8)	C12—C13—C14—C15	−0.29 (14)
C1—C6—C7—N1	4.84 (13)	C12—C13—C14—N6	178.83 (8)
C5—C6—C7—C17	5.55 (14)	O3—N6—C14—C13	−2.62 (14)
C1—C6—C7—C17	−173.42 (9)	O2—N6—C14—C13	176.82 (10)
N1—N2—C8—O1	−4.64 (14)	O3—N6—C14—C15	176.54 (10)
N1—N2—C8—C9	175.17 (8)	O2—N6—C14—C15	−4.02 (14)
N4—N3—C9—C10	−0.25 (11)	C13—C14—C15—C16	1.63 (15)
N4—N3—C9—C8	−178.64 (8)	N6—C14—C15—C16	−177.48 (8)
O1—C8—C9—N3	−169.32 (9)	C14—C15—C16—C11	−1.05 (14)
N2—C8—C9—N3	10.87 (12)	C12—C11—C16—C15	−0.83 (14)
O1—C8—C9—C10	12.66 (16)	N5—C11—C16—C15	175.08 (8)

### Hydrogen-bond geometry (Å, º)

**Table:**

*D*—H···*A*	*D*—H	H···*A*	*D*···*A*	*D*—H···*A*
C13—H13*A*···O1^i^	0.95	2.32	3.2226 (12)	158

Symmetry code: (i) *x*, *y*−1, *z*.
